# Centrioles Position the Nucleus and One Another

**DOI:** 10.1371/journal.pbio.0050161

**Published:** 2007-05-22

**Authors:** Richard Robinson

Centrioles are among the simplest structures within the eukaryotic cell, but they play a surprisingly complex set of roles. They are composed of nine parallel protein rods, which form ribbed, hollow tubes with more than a passing resemblance to microscopic rigatoni. In pairs, centrioles form the heart of the centrosome, which hovers near the nucleus and coordinates chromosome division during mitosis. Singly, and docked to the plasma membrane, they dictate the position and growth of flagella and cilia, the hair-like extensions whose coordinated beatings can either move the cell through liquid or move liquid across the cell’s surface. They replicate themselves by a templating process, without any direct instruction from the cell’s nucleus.

In a new study, Jessica Feldman, Stefan Geimer, and Wallace Marshall reduce some of the mystery but none of the fascination surrounding centrioles by revealing that daughter centrioles rely on mother centrioles to find their proper spot in the cell, and that together, the centrioles play a crucial role in positioning the nucleus and other cell organelles.

The authors chose to study the unicellular alga, *Chlamydomonas reinhardtii*, whose oblong cells sprout two flagella at one end (called the apex). They began by developing 10,000 mutant cell lines and inquired which had defects in centriole positioning. *Chlamydomonas* cells contain a photosensitive “eyespot,” which coordinates flagellar movements that move the cell toward the light. This relies on a fixed orientation of eyespot and flagella (and thus centriole); mutants with improper centriole position could be discovered by their inability to swim to the light.

They identified 13 centriole mispositioning mutants, collectively dubbed *asq* (“askew”) mutants. In these cells, the putative apical centrioles were found up to 60 degrees away from the apex, and the position varied widely from cell to cell (in unmutated cells, most centrioles were found between 10 and 30 degrees away from the apex).

Older centrioles give rise to younger ones by a process of direct replication, and daughters remain tethered to mothers by fibers. Of the 13 mutants, nine retained this attachment, and each of these cells included only two flagella, improperly positioned in relation to the cell as a whole, but correlated with each other. In contrast, in four mutants, mother and daughter separated, and the flagella positions were no longer correlated. Furthermore, because they were no longer attached, the centrioles did not separate properly during mitosis, and so the number of centrioles per cell varied over the generations. As a result, offspring cells developed variable numbers of flagella, from a low of zero to a high of seven.

The sundering of the connection between mother and daughter allowed the authors to ask other questions about the relationship of these two centrioles. Mother centrioles bear fine structural details not found on the daughter, and the authors hypothesized these might help direct the mother to the proper position on membrane; ordinarily, they proposed, the daughter would tag along and be positioned by her relation to the mother. In support of this model, they found that in *asq2* mutants, mother centrioles were more often located properly than daughters.

**Figure:**
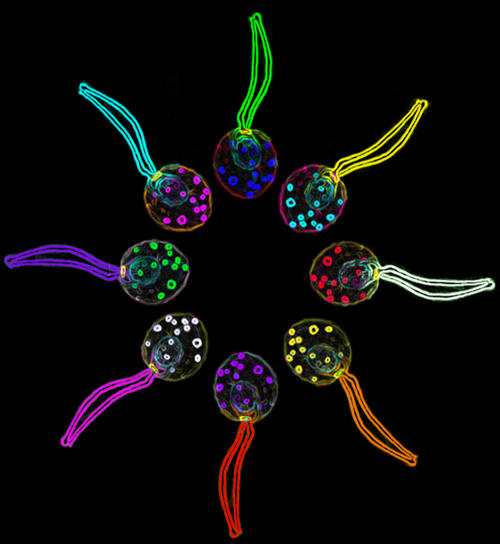
Chlamydomonas cells with flagella, plasma membrane, nucleus, and chloroplast nucleoids highlighted in neon. (Image: Jessica Feldman)

Flagella are not the only structures that get out of place when centriole connections are cut. In cells mutated for centriolar–nuclear connections, the nucleus also wandered. But the centrioles themselves remained in the correct position in these mutants, suggesting that in a normal cell, it is the centrioles that control the position of the nucleus, and not vice versa. Some other structures, including a contractile vacuole, also wandered free without centriolar instructions.

Finally, the authors examined other mutants for structural features of the centriole that may be involved in positioning. Mispositioning was greatest in those missing the distal portions of the centriole, suggesting this region is crucial for docking onto the apical membrane.

The identification of these mutants will allow researchers to ask further questions about centriole function and the relation of centrioles to each other and to other cell structures. Both the present study and future ones will also allow progress to be made in understanding how cilia on human cells, such as those lining the lung passages, develop and maintain their coordinated motion throughout the life of the cell and pass that trait on to their cellular offspring. Defects in this ciliar coordination underlie several human diseases, suggesting that further exploration of the mysteries of the centriole may have practical applications as well.

